# Effects of azithromycin on serum inflammatory factors and T lymphocyte subsets in patients with gynecological mycoplasma infection

**DOI:** 10.12669/pjms.38.8.6283

**Published:** 2022

**Authors:** Mengdi She, Cheng Chen

**Affiliations:** 1 Mengdi She, Department of Pharmacy, Changxing People’s Hospital, Huzhou 313100, Zhejiang Province, P.R. China; 2 Cheng Chen, Department of Gynecology, The First People’s Hospital of Huzhou, First affiliated Hospital of Huzhou University, Huzhou 313100, Zhejiang Province, P.R. China

**Keywords:** Gynecological mycoplasma infection, Azithromycin, Inflammatory factors, T lymphocyte subsets

## Abstract

**Objectives:**

To investigate the effect of azithromycin on the levels of serum inflammatory factors and T lymphocyte subsets in patients with gynecological mycoplasma infection.

**Methods:**

Records of 250 patients with gynecological mycoplasma infection, treated in our hospital from May 2020 to June 2021, were retrospectively divided into two groups based on the received treatment. Patient (n=120) that were treated with levofloxacin tablets comprised the control group, and patients (n=130) treated with levofloxacin tablets (250mg) and azithromycin tablets comprised the observation group. Changes of serum inflammatory factors and T lymphocyte subsets in the two groups after two weeks of treatment with corresponding drugs were analyzed retrospectively.

**Results:**

After the treatment, the levels of C-reactive protein(CRP), interleukin-6(IL-6) and tumor necrosis factor α Tumor necrosis factor α TNF- α in the observation group were significantly lower than those in the control group (P<0.05). Levels of CD3+, CD4+ and CD4+/CD8+ in the observation group were lower than those in the control group (P<0.05). The total clinical efficacy in the observation group was 93.08%, significantly higher than 80.00% in the control group, and the total incidence of adverse drug reactions was 6.15%, significantly lower than 14.17% in the control group (P<0.05).

**Conclusion:**

Azithromycin used in clinical treatment of patients with gynecological mycoplasma infection can effectively improve anti-inflammatory and immune response, improve the clinical efficacy of the treatment and reduce adverse reactions.

## INTRODUCTION

Female mycoplasma infection is a common gynecological inflammatory sexually transmitted disease that causes inflammatory reactions in cervix, vagina and other parts of female reproductive tract.^1^ The clinical symptoms of patients with gynecological mycoplasma infection include obvious discomfort in the reproductive tract, increased vaginal secretions, pain during urination etc. After the onset of the infection, lack of timely treatment may lead to aggravation of the disease that may progress into salpingitis, pelvic inflammation, endometritis, etc., and even affect patient’s reproductive function.^2^ The main pathogens causing female mycoplasma infection are human mycoplasma and Ureaplasma urealyticum. Mycoplasma is the smallest microorganism that exists outside the cell.^3^ At this stage, patients with gynecological mycoplasma infection are mainly treated by antibiotics, such as Acetylspiramycin and levofloxacin. However, clinical research and practice results show that when levofloxacin and other drugs are used alone, the course of treatment is long, and the long-term use of drugs can cause a variety of adverse reactions that have a significant impact on the treatment compliance of patients, and affect the overall control effect of the disease.^4^,^5^ Azithromycin is a kind of macrolide antibiotics, which has shown good effectiveness in the treatment of many diseases caused by sensitive bacterial infection.^6^ This study focuses on the role of azithromycin in inflammation control and immune function improvement in the clinical treatment of patients with gynecological mycoplasma infection, in order to provide more valuable reference for the clinical treatment of the disease.

## METHODS

Medical records of patients, diagnosed with gynecological mycoplasma infection treated from May 2020 to June 2021 were retrospectively analyzed. The ethics committee of our hospital approved this study (Approval number: HZCX-L210521, May 18^th^ 2021. A total of 250 patients were included in this study, with ages ranging between 21 and 56 years, an average of (37.09±8.17) years.

### Inclusion criteria:

- meet the diagnostic criteria related to female mycoplasma infection,^7^ such as positivity for Mycoplasma hominis, genital Chlamydia trachomatis and Ureaplasma urealyticum in the cervical secretions;
- clinical symptoms such as urethral tingling, vulvar pruritus, increased leucorrhea and turbidity;
- complete relevant clinical data of the diagnosis and treatment

### Exclusion criteria:

- accompanied by other gynecological infectious diseases;
- serious cardiovascular and cerebrovascular diseases or liver and kidney dysfunction;
- contraindications to the drugs used in the study;
- use of other reproductive tract drugs or other antibiotics; mental illness.

All processes of this retrospective analysis were in full compliance with the relevant rules and regulations of the medical ethics committee of our hospital. Patients were retrospectively divided into two groups based on the received treatment. Patients (120) that were treated with levofloxacin tablets (250mg) only comprised the control group. Patients (130) that were treated with both levofloxacin and azithromycin comprised the observation group.

### Treatment with levofloxacin tablets:

The patient was treated with levofloxacin tablets (250mg) (Zhejiang Puli Pharmaceutical Co., Ltd., H20213082, specification: 0.25g*30 tablets) in warm water once a day, one tablet per time for two weeks.

### Levofloxacin and azithromycin treatment:

The patient was treated with levofloxacin tablets and azithromycin tablets (Pfizer Pharmaceutical Co., Ltd., H10960167, specification: 250mg*6 tablets). The application method of levofloxacin tablets is the same as above. Azithromycin tablets were used as follows: 0.5g/time, once a day on the first day; Low 2~5D oral drug 0.25g/time, once a day, all taken two hour after meals. Drugs were used continuously for two weeks.

During the treatment, all patients were prohibited from bath and sexual activities and were instructed to follow good personal hygiene and diet guidelines. The effect of the treatment was evaluated one week after the end of the antibiotic regiment. All the patients, included in the study, had medical records of the basic information such as age and course of disease during treatment, and all the relevant indexes collected after two weeks of drug treatment. The following indexes were analyzed:

- Serum inflammatory factor levels before and after treatment (one week after stopping medication). Detection methods: 3ml of fasting venous blood in the morning was extracted and centrifuged, and then the ELISA kit provided by Nanjing Jiancheng Biotechnology Co., Ltd. Was used to detect the level of relevant inflammatory factors according to the reagent instructions. The specific measurement indicators are tumor necrosis factor α(Tumor necrosis factor α TNF-α), Interleukin-10(IL-10) and C-reactive protein (CRP).
- T lymphocyte subsets of patients before and after treatment (one week after stopping medication). Detection methods: 3ml peripheral venous blood was collected and placed in a special anticoagulant test tube, and then the levels of relevant T lymphocyte subsets indexes (CD3+, CD4+, CD8+ and CD4+/CD8+) were detected by flow cytometry (epics elite, USA)
- Curative effect included one of the following categories: ***Recovery:*** the pathogen examination result is negative after treatment, and the relevant clinical signs and symptoms completely disappear.

### Significant effect:

the pathogen examination result is negative, and the clinical signs and symptoms are significantly improved compared with those before treatment.

### Effective:

the clinical signs and symptoms are significantly improved compared with those before treatment, but the pathogen examination result is positive. *Ineffective*: after treatment the clinical signs and symptoms were not improved or further aggravated.^8^ Total efficacy was calculated as a ratio of (cured + markedly effective + effective)/total number of cases×100%.

### Safety of drug Treatment:

Records of all the adverse reactions after drug treatment were noted.

### Statistical analysis:

Statistical analysis and processing were performed by SPSS 22.0 software. All measurement data are expressed in (x̄±s) for t-test; The counting data is expressed in percentage “n (%)” and is calculated χ^2^ Inspection and treatment. P<0.05 was considered statistically significant.

## RESULTS

A total of 250 patients met the inclusion criteria of the study and were retrospectively divided into two groups based on the treatment they received. Of them, 120 patients were treated with levofloxacin tablets orally, and 130 patients were treated with levofloxacin tablets and azithromycin tablets orally. There was no significant difference in age, course of the disease and other related basic data between the two groups (P>0.05) (Table-I). There was no significant difference in the levels of IL-6, CRP and TNF-α between the two groups before treatment (P>0.05). After two weeks of corresponding drug treatment, IL-6, CRP and TNF-α levels in the levofloxacin tablets and azithromycin (observation) group were significantly lower than those before treatment and significantly lower than those in the levofloxacin only (control) group (P<0.05) (Table-II). There was no significant difference in the measurement results of several indexes of T lymphocyte subsets between the two groups before treatment (P>0.05). After two weeks of treatment with corresponding drugs, CD3+, CD4+and CD4+/CD8+in the observation group were significantly higher than those in the control group (P<0.05) (Table-III). The total efficacy of the observation group was higher than that of the control group, and the total incidence of adverse reactions was lower, the difference was statistically significant (P<0.05) (Table-IV).

**Table-I:** Comparison of basic data of the two groups of patients.

Group	n	Age (year, x̄ ±s)	Course of disease (months, x̄ ±s)	Type of disease		

			Salpingitis	Pelvic inflammatory disease	Endometritis
Control group	120	21~53 (37.00±8.09)	0.5~12.00 (4.87±2.78)	60(50.00)	44(36.67)	16(13.33)
Observation group	130	22~54 (37.17±8.27)	0.5~12.00 (.5.27±2.87)	65(50.00)	48(36.92)	17(13.08)
*χ^2^*/*t*	-	0.163	1.116		0.004	
*P*	-	0.870	0.266		0.998	

**Table-II:** Comparison of CRP, IL-6 and TNF-α levels before and after treatment between the two groups (x̄ ±s).

Group	n	CRP (mg/L)		IL-6 (pg/ml)		TNF-α (pg/ml)	

		Before treatment	After treatment	Before treatment	After treatment	Before treatment	After treatment
Control group	120	45.22±4.64	37.23±3.39	47.26±4.20	24.55±3.26	44.60±4.29	36.76±3.50
Observation group	130	46.11±4.56	25.82±3.46	48.25±4.28	16.17±3.78	44.79±4.49	19.15±3.73
t	-	1.516	26.302	1.841	18.826	0.345	38.442
P	-	0.131	<0.001	0.067	<0.001	0.730	<0.001

**Table-III:** Comparison of CD3+, CD4+ and CD4+/CD8+ measurement results between the two groups before and after treatment.

Group	Time	CD3^+^(%)	CD^4+^(%)	CD^8+^(%)	CD^4+^/CD^8+^(%)
Control group	Before therapy	53.62±3.92	35.85±3.11	27.25±3.05	1.30±0.08
Observation group		53.27±3.99	34.50±2.72	27.82±2.11	1.31±0.07
Test value	t	0.695	1.331	1.716	0.358
	P	0.488	0.186	0.088	0.721
Control group	After treatment	55.03±3.53	36.45±2.70	27.41±2.24	1.33±0.08
Observation group		63.72±3.41	41.01±3.89	27.55±2.16	1.49±0.17
Test value	t	19.776	10.822	0.492	9.658
	P	<0.001	<0.001	0.623	<0.001

**Table-IV:** Comparison of clinical total effective rates and adverse reactions between the two groups (Example).

Group	n	Total efficacy					Adverse reaction				

		Recovery	Remarkable effect	Effective	Invalid	Total effective rate	Dizzy	Nausea	Vomit	Diarrhea	Total
Control group	120	29	43	24	24	80.00%	6	6	3	2	17(14.17)
Observation group	130	56	52	13	9	93.08%	3	3	1	1	8(6.15)
χ^2^	-					9.313					4.452
P	-					0.002					0.035

## DISCUSSION

The results of this retrospective study showed that a combination of levofloxacin and azithromycin was associated with significantly lower levels of the related inflammatory factors (TNF- αThe levels of CRP and IL-6). Addition of azithromycin to the routine treatment regimen can significantly improve the efficiency of inflammation control, clinical signs and symptoms in patients with gynecological mycoplasma infection. Our results are in agreement with the study of Madeleine EO et al.^9^ that showed that azithromycin treatment significantly reduced relevant inflammatory factors in patients with viral infection (compared with those before treatment), as well as improved overall relevant signs and symptoms caused by infection. Levofloxacin is a commonly used quinolone antibiotic. It has the advantages of wide antibacterial spectrum and strong effect.^10^ At present, levofloxacin is widely used in a variety of bacterial infection diseases and shows good application effect. However, when used alone, the course of treatment is long, which may cause adverse reactions. Moreover, its effect on some Staphylococcus and Chlamydia needs to be further improved.^11^ Azithromycin is a type of macrolide antibiotics also commonly used in clinical practice. It also has strong antibacterial effect and wide antibacterial spectrum. Azithromycin shows strong inhibition on 98% of anaerobic bacteria and Mycoplasma.^12^,^13^ At the same time, the physiological utilization of this drug is greater than 55%, T_1/2_ is relatively longer, and high drug concentration can be obtained after local use. Therefore, the anti-inflammatory and antibacterial effects are significantly higher than other antibiotics.^14^,^15^ The results of this study showed that the improvement effect of T lymphocyte subsets (CD3+, CD4+and CD4+/CD8+) in the patients who were treated with the combination of levofloxacin and azithromycin was significantly better than that in the control group (levofloxacin alone). The application of azithromycin also shows a good regulation of the immune function, which may help to achieve better clinical effectiveness and reduce the risk of recurrence. Cramer CL et al.^16^ reported that azithromycin showed good immune regulation in the clinical treatment of patients with respiratory diseases. That in turn helped to improve the overall immune function of patients, contributing to the improvement of patients’ condition, recurrence control and prognosis. Azithromycin is used in the treatment of patients with gynecological mycoplasma infection. After entering the human body orally, azithromycin can be quickly and widely distributed throughout the body, and can combine with the 50S ribosomal subunit of pathogenic sensitive bacteria, so as to affect the synthesis of bacterial protein, exert bactericidal effect and regulate the immune function of the body.^17^ At the same time, azithromycin also plays a strong anti-inflammatory and bactericidal role to reduce cell damage, and to improve the total effective rate of clinical treatment.^18^ The application of azithromycin can maintain the effective concentration in tissue and blood for a long time, play a strong and long-term anti-inflammatory, bactericidal and anti-inflammatory role, reduce the dosage of other antibiotics or shorten their use time, thus reducing the rate of adverse drug reactions. Our study shows that the total clinical efficacy of the observation group was significantly higher than that of the control group, and the total incidence of adverse reactions is significantly lower.

### Limitations of the study:

In addition to being a retrospective study, this is single center study with a small sample size and no long-term follow-up observation. Further multi-center, large sample- and long-term follow-up studies are, therefore, needed.

## CONCLUSION

Azithromycin in combination with levofloxacin in the clinical treatment of patients with gynecological mycoplasma infection, is associated with the improved anti-inflammatory response, promotes the effective regulation of T lymphocyte subsets, and improves the effectiveness and safety of clinical treatment.

### Authors’ contributions:

**MS:** Conceived and designed the study.

**MS & CC:** Collected the data and performed the analysis.

**MS:** Was involved in the writing of the manuscript and is responsible for the integrity of the study.

All authors have read and approved the final manuscript.
